# A Medico-Legal Review of the Case of Patrick Maude; Who Was Convicted for Murder and Executed at Newark, N. J.

**Published:** 1860-04

**Authors:** Charles F. J. Lehlbach

**Affiliations:** New York


### SELECTIONS.

### *A Medico-Legal Review of the Case of Patrick Maude; who was convicted for murder and executed at Newark, N. J.—The Plea of Insanity.* By Charles F. J. Lehlbach, M. D., of New York.

[Continued from page 433.]

Maude insisted on conducting his own trial, during the whole of which he protested against the proceedings of council appointed for him by the court, looking upon them as hirelings of his conspirators, and their plea of insanity for him, as a gross outrage upon him, prompted by his supposed enemies. As specimens of his psychical condition, we make the following extracts from the report of the trial, preserving the order of their occurrence; they will show:

5. *Insanity in his general conduct.*

"W. L. Meeker was the first Juror called, and on taking the stand, was asked by Maude if he was a citizen of Newark.

Juror—Yes.

Maude—how long have you resided here?

Juror—Forty years.

The prisoner then proceeded to question the juror further, when he was stopped by Judge Ogden, who told him he had the right to challenge, but not to question the jurors.

Maude—No man can sit on my jury who has taken part in or has heard of an inquisition held on my death by Father Callan, a Catholic priest.—This inquisition was held, but it is unconstitutional and has made me a proscribed man.

The Judge endeavored to explain to the prisoner his rights in reference to challenging persons.

Mr. Gifford said the prisoner desired to question the jurors in reference to their cognizance of the affair.

Maude—Mr. Gifford, I have my own case in hand, and I want this question answered fairly. I am a proscribed man, and no longer an American citizen—therefore I have returned my naturalization papers."

"Mr. F. H. Teese, one of the counsel, having asked a question of a juror, Maude said—'You are no counsel of mine, Mr. Teese.'

Maude said his object in questioning the jurors had been to show that the inquisition to which he alluded had been held contrary to the Constitution.

The jury being all sworn, C. Parker, Esq., opened the case on the part of the State. He said the prisoner at the bar was indicted for the murder of Mary Turbett, on the 28th of May last, and he then proceeded to define the different degrees of murder, and commenced to review the evidence to be produced in this case. He alluded to the fact that Maude had threatened to take the life of his sister and wife, and two others, (*Maude interrupting—'Connected with the inquisition say,'*) officers of the State. He traced Maude from the Lunatic Asylum, after his escape therefrom, to this city, reviewing the details of the tragedy, and the arrest of the offender. He then proceeded to speak of the decisions in relation to the plea of insanity being made available—when he was interrupted by Maude, who said, 'You need not read that Mr. Parker, I will not go on the plea of insanity for you nor your jury. Mr. Parker read the law on the subject, and concluded by stating the reasons for the presence of the Attorney-General."

We next come to the opening by the defence.

"The defence was opened by Frederick H. Teese, Esq., who said he was counsel for a prisoner accused of murder, (here Maude said 'you are not my counsel,") and one who would not communicate with him. He remarked that the prisoner was a strange man—not to say insane. It was the duty of the jury in order to convict him, to find that Mary Turbett came to her death by a pistol shot from the prisoner. If they should find that he was the means of her death, they should acquit him on the plea of insanity. (Maude said: 'I will not allow the plea of insanity—it is a conspiracy.') Mr. Teese referred to the case of Spencer, and read a decision relative to insanity. He should try the case according to law. (Here he was interrupted by Maude who said: 'You are not proceeding according to law.') The State, Mr. T. continued, claimed that Patrick Maude had fled from the lunatic asylum, come to Newark and killed his sister. Under the same circumstances, had he made a will and died, the Court would render it null and void, claiming that he was insane. Now the same law would necessarily be brought to bear in this case. Persons under the influence of insanity very often thought their nearest relatives and friends had conspired against them, and though conscious of the crime of murder, they were impelled to kill them. He referred to several cases in England and this country illustrating this. In judgment, he said, the true question to be answered by the jury is: 'Was the prisoner insane or not at the time he committed the deed.'

"The prisoner was once a man well to do in the world. About seven or eight years ago he lost his property.* Brooding over his losses he had

* This needs explanation. A gentleman, perfectly conversant with the matter, states to us that it was simply thus: When Maude had married, his wife owned a house worth about $3,000, upon which there was a large mortgage. Maude subsequently went to work in Pennsylvania, coming home once or twice a month, sometimes oftener. He took to drinking, and did not save his money, quarrelled when at home, and often destroyed furniture, etc., about the house. His wife, who kept a store, was unable to pay interest on the mortgage, a forestallment finally took place, and the house was sold. This is the whole real foundation to his insane idea about the loss of his property, the conspiracy, the inquisition, the witchcraft, etc. L.

become insane on the subject, and concluded that his wife, sisters and nearest friends had conspired against him. (Maude said, 'How do you know?') He made an assault with intent to kill, on John Connelly, and on being tried was found to be insane, accordingly sent to the lunatic asylum. On getting out he thinks an inquisition has been held against him by Father Callan, Judge Haines, Mr. Parker, and his wife and deceased sister. An inquisition was held, composed of several physicians in order to remove him to the asylum—but not a Spanish inquisition, as his insane mind had supposed it to be. Since that time he has raved on this inquisition or conspiracy, as he thinks it was. Mr. T. referred to the case of Margaret Garrity, who was insane, yet never had given half the proof of it as in the present case. Maude is an insane man in every sense of the word (Maude here said, 'Prove it if you can,') and I leave it for you to do him justice.

"During the whole of Mr. Teese's remarks he was interrupted by Maude, who repudiated the idea of being sent to the lunatic asylum."

We omit the testimony of various witnesses whom Maude cross examined in a similar manner as he did the jurors, and constantly interrupting the proceedings.

Next follows Maude's speech on his defence, which is by some looked upon as a strong proof of his sanity, because he succeeded in not introducing his hallucinations into it, argued the legal points with apparent logic, and especially because in it he plead so earnestly in behalf of the soundness of his mind.

"REMAKS OF MAUDE,—Maude then arose and said that he wished to make a statement to the jury. He said this trial was one for life or death, and the question of sanity or insanity had nothing to do with it. The jury were to decide him guilty or not guilty of the wilful murder of Mary Turbett. If there is evidence enough to warrant you in finding a verdict of guilty do not perjure yourselves. If there is not evidence enough, the verdict must be, not guilty. There is no half way house between the two —it must be either the one or the other. The prisoner then alluded to his challenging the jurors, and said he believed they were competent men or they would not have been selected. He then commenced to review the evidence. Dr. Dodd had testified that the bullet found during the post-mortem examination was conical, which I take to mean that it was in shape like a heart. The other doctor said the bullet was round and produced it in Court. It makes no difference what was the shape of the bullet, whether it was a square bullet or triangular, so long as it caused death; but it makes a difference in the evidence. Dr. Dodd also stated that on the morning of the 29th of May he was at the station house in conversation with several gentlemen, and he said that the Hon. Judge Waugh, on that occasion, made the remark that Courtlandt Parker, had acted very wrong in sending me to the lunatic asylum, that he ought to have sent me to prison until I became insane, and then sent me to the asylum. The first witness called, I believe, was Patrick English, who testified that he saw a man on the night of the death of Mrs Turbett in Dowing street, and that he wore dark clothes and a straw hat. Many people dress in that manner. He saw the man running but he could not say it was me. About the same time he saw a colored man who appeared to come out of Turbett's store, and after going a short distance with him he returned to the store and found it in darkness, and said that Mr. Turbett was then looking for a light. I borded in that house some two years and I know the position of the place exactly. (Maude then described the arrangement of the house and store, the location of the doors, &c.) One witness said that all the doors were open at the time the shots were fired. The man who shot Mrs. Turbett, no matter who he was, could have stood in the parlor as well as at any other point. From the parlor he could even hit the beer pump near which it is testified that she was standing. Mr. Abraham Turbett was the first man found in the store after the occurrence, and yet he has not been summoned here as a witness. Why is this? The prisoner then reviewed the evidence of Mrs. Haywood. She was present in the store when the shot was fired, yet she saw no person fire it. It must have been fired in the house because there were no windows broken. Mrs. Lewis said she saw a man step from the stoop, and that he was dressed in dark clothes, but although she testified that he passed, I believe, within three feet of her, she did not know him. The rest of her testimony in relation to the manner in which the body was found, it is not necessary to allude to. Mr. Jones is the only witness who has testified to seeing me at that time. He states that himself and two or three others were standing on the cornor of Jefferson and Market streets, when a tall man in dark clothes and straw hat came up and caught him by the arm and said 'boys take me to the depot, or show me to the depot,' and after the man had passed he exclaimed there is Patrick Maude and he ought to be arrested, for he had escaped from the lunatic asylum and his poor wife was hid from him for these two days. In his evidence he said he had known me for ten years. That is true, and perhaps for a longer period. Now, is it likely that I, having known him for so long a time, would come up to him and say, 'Boys take me to the depot.' I believe, I know the way to the depot as well as any one in Newark. This witness testified that he had never heard anything derogatory to my character except what had been said to him by my wife. He admitted that he rented a pew in the church in partnership with her. I make no comment on the evidence. Patrick Ravey testified also. I knew him very well, have gone to his store frequently when I resided here. He testified that he arrested me, and that I was walking leisurely along at the time in Commerce street, within fifty yards of Mulberry street. The constable that was with him said when he first saw me I appeared to be emerging from a hiding place like a murderer, and that I seemed to be trying to skulk away, and look which way I would run. Ravey wore his own coat but the constable wanted his epauletts on. He then reviewed the evidence of police officer Stanley, who found the pistol in the yard of the Gas Works, but did not know where such works were situated. He said one barrel of the pistol was cocked, but not loaded, and that he did not know whether the other barrel was loaded or not. Then the man from New York who sold a pistol to a man he thought was me, did not state that the pistol produced here was the one he sold, and said the man that bought the pistol had a beard. It was the rule in the asylum to shave the inmates twice a week, and in some of the halls three times. Where I was during the latter part of my confinement they shaved three times a week. Therefore I could not have had any whiskers when I escaped. I have not been shaved now in four months, and consequently did not appear as it was said as outlandish as I do now. Outlandish is not an American phrase, neither is it an English one. It is an Irish slang word. I do not want to run down my country, but their are bad men of all countries, probably this man learned that phrase before he came over. The prisoner then alluded to his first trial, and the fact that he had been declared by Dr. Dodd to be perfectly sane on all points but one. The chief of the slaughter house (asylum) had alluded to a stone tied in a handkerchief that I had to use while there (our reporter could not understand at this point whether the prisoner said that he had struck any one with the stone or not.) In the fall of 1858, when at the asylum, I was allowed to walk every day for an hour or so in an outer hall and while there on one occasion, I had some hickory nuts. I took a stone in my cell with me to crack them on the window sill. This stone remained in my cell for a long period. On the first day of January, which was a Saturday, I believe, I refused to bathe in cold water, it being the custom for all to bathe. The water was bitter cold, and I would not endanger my health by bathing in it. On the 8th of the same month, which was also Saturday, I refused again, and when they attempted to force me I used this stone, tied in a handkerchief, in self-defence, though I did not strike any person with it. I was put in the hall among the maniacs, who were in there native skins. I was possessed of my senses and I declare truly that I did not sleep 48 hours out of the whole time I was there, some two or three months. I was no insane man, and am none, but they were determined to make me insane. I do not go on the plea of insanity. I don't want it adduced in my behalf. I must be cleared, if cleared at all, on other evidence. Insanity has nothing to do with the case. If I am to be tried as to sanity it must be at a separate trial; the two should not be mixed together. If you are satisfied from the evidence that I am the man who committed this crime, then you must bring in your verdict of guilty, but if the evidence is insufficient you must make your verdict 'not guilty.' It is no murder in the 2d degree. It is either to be wilful murder or nothing. I have not read Kent and Blackstone, but I believe the law on this is as I state it. There is no evidence to prove that I was seen in the house, by the house or in the street when the murder was committed, with the exception of Jones, who says I have known him for ten years, and that I addressed the party he was in as "boys." I will not detain you with any remarks; I have merely gone over the evidence as it has been produced here. Gentlemen of the Jury, I leave the case in your hands exactly as it is. You will find a verdict of guilty or not guilty, just as you believe the evidence to warrant. Do not perjure yourselves. Do justice in the eyes of God and the world."

The prisoner delivered the foregoing remarks in a clear voice, and spoke slowly and composedly. There was no excitement in his manner and actions.

Compare however his speech after the conviction, before receiving his sentence, when the dread of being pronounced "not guilty on the plea of insanity," was no longer before him, forcing him to restrain and conceal as far as lay in his power, the true condition of his mind:

"Though it will make little difference what I say, I will speak, for this is an execution, or rather a crucifixion. My child was sacrificed and murdered by Mr. Turbett, Mrs Turbett, my damned wife, and others. This is what caused my first assault on my villian wife. She and Courtlandt Parker, the prosecuting attorney, made the trick against me; he was a Pharoah, and now wants to make me a Pontius Pilate. An inquisition was held on me by Parker and Farther Callen. I am tortured by witchcraft from my damned family. All the insane maniacs in asylums are made so by witchcraft. I'll ask no pardon, and will accept none from Governor or President. I've done no wrong; what I did was in self-defence. I was brought here before for the first offence in my life. I plead guilty, but got no mercy. I was sent to prison as an insane man, for life. I say here, I'm not insane, you can execute me, but I'm not insane. Charley Gifford was my counsel, but he, while addressing the jury received something on his back, which made him say to the jury, 'he must be convicted.' All I ask is, that my body may not be given to my relatives, for they are devils; nor to the doctors for dissection. In passing sentence do not pray for me; I pray to heaven for vengeance. Now pass your sentence."

Not content with making the above speech, he frequently interrupted the Judge while he was passing sentence, with such expressions as the following: "You needn't go over the evidence." "Witchcraft is against the law of God and man." On Judge Ogden concluding the sentence, Maude glaring fierecely and with set teeth said, "'Don't pray for me. I pray for vengeance to Jesus Christ. I'll allow no priest or clergyman to come near me, I'll maim him if he does."

And now the last articulate outburst of his diseased brain; his remarks to the jailor, on observing the scaffold, on Thursday morning:

"You need not be afraid of me for you cannot bully me down by the sight of a gallows. I prefer to be executed, for I have suffered everything but death in that cell. I have suffered more than death through my family by witchcraft. This morning, when I awoke—for I slept well last night—I saw my wife, who tormented me, as well as Turbett, who recieves a weekly salary from Father Callen and Courtlandt Parker to torment me, through witchcraft. Jesus Christ was tormented by the Jews, the Gentiles, by Pharaoh, and I am by Turbett, his wife, and that d—d witch that lives in Van Buren street. When Turbett came to this country, I supported him and his wife for months, till they got $600 from Father Callen, and $750 from Courtlandt Parker, which makes $1,350, to condemn me, for which I am to be executed to-day.

"The Jury perjured themselves. So did the witnesses, and they have robbed me through their damned witcheraft. Three years ago they murdered my child, as sweet a child as there was in America. When I returned with my horse and buggy—for I did not know it then—did not Turbet take the child by its little hand, and with the other grasp it over its mouth and plunge it in a crock of water and smother it? The marks were on its face. Don't they persecute me all the time with their damned witchcraft? I have peace with God through Jesus Christ, for I suffer as a martyr through the damned witchcraft. Let it go through England that America is ruled by witchcraft; when the prisoners leave here, all that can must leave the country, for you all are the victims of witchcraft. It has been told me so."

Then the speech, immediately before his execution, of which the following is but a poor synopsis:

"Now, you all have assembled together here to witness an American crucifixion, though you can call it an execution. I was placed a prisoner here, not by the law of your land, but by the Pope, by your Popish priests, and by infernal withchcraft. Turbett's wife was bound to destroy me; she was backed by the priests and the whole Popish Church. What is that Church? What is its history? I will tell you something about it, if I can have time." (Turning to the Sheriff, he said: 'Are you going to give me time?' and received an affirmative answer.) "I'll let you see I'm no fool, and am not ignorant, though a poor hard-working man. I've read history and poetry; I began with Pultarch's Lives, and read all about Romulus and Remus, and next about Julius Cæsar, and a greater man than all the rest, namely, Dioganeus, (Diogenes.) I read, too, about Charle-magene. Rome was once a great city. It was greatest in the days of Julius Cæsar, but 300 years afterwards it submitted to the Pope. Yes, *submit* is the word, and that's what you're all doing here. You are all slaves—black and white. The Bible was made, like a lawyers's indictment, with every other line framed to slaughter men. One Bible was first gotten up; then another and different one was made by the Council of Nice; then another was made at Trent. There are three Bibles—which of them is true? Then, in the fifteenth century, the reform started up. All the great reformers were heads of the Catholic Church, who were mad because they couldn't be Popes. There were Luther, Calvin, and Knox; each one made a Bible—that makes six Bibles. Then there was Henry the Eighth, he started a reform in 1512. Oh, we read Goldsmith and Hume? I'll let you see I'm not a fool. Henry was driven on by witchcraft—just as I am. I am impelled by witchcraft, just as the steamer is driven through the water. Henry the Eighth's minister, Cardinal Wolsey, was the greatest man of his time, and the most powerful, except Philip of Spain. When he wanted to be Pope, Philip wouldn't let him. He couldn't revenge himself on Philip, so he murdered Philip's sister—Catharine of Aragon. I stood in my cell, the other day, and asked an old Dutch patriarch, who came to see me, whether all this was not true? I asked him how they could say 'improved and revised' on the title-page of the Bible, if it was God's word in the first place? He said: 'I know it, Patrick; they all make mistakes. You ish a martyr. God help you!"

"Until the seventeenth century there were no poor-houses and charter-asylums in the old country, no paying tithes to priests. They were better off then. Now everthing belongs to the necromancers. The priests and the law drive the landlord and the laborer to the ale-house. All the agents were lawyers, doctors, and priests. The working-men used to have snug little houses, and few were transported, or had to come to America. Then the necromancers came in and ruined everybody. Then poor people took to thieving and crime. You are all slaves—whether in or out of jail; all damned by the rule of necromancy, and lawyers, doctors, and priests." (At this juncture loud shouts and laughter were heard outside the jail.) "I landed in New York. Come up here, Bishop Hughes! Come up here, Henry Beecher Stowe, who wrote Uncle Tom's Cabin! You are both heads of churches. Is there any man from Kentucky here?—if so, come up, and I will count slaves against you. Take the poor-houses and prisons on Blackwell's Island, and the asylums—which ought to be called slaughter-houses—and your Connecticut factories. These are full of your northern slaves. Come on you d—d necromancers! you can make little by executing me. Don't think that rum-drinking or religious excitement fills your asylums. It is done by witchcraft—by lawyers, doctors, and priests—those three engines of Satan. They have women for their machines. The women are not so much to blame. They are spoiled by priests in their childhood. Then the doctors help them to produce abortions and wholesale murders. They kill their fathers, mothers and brothers, and drive men to suicide and asylums. I'm telling you the truth about your country. They shut up rich men for their property. Everybody wants an office in this country. The professional robber is a fine gentleman. Here they butcher men like hogs. The poor laborer is led by d—d prostitutes to take rum, and then shut up for life. Europeans, clear the country! Go home to Europe, where your life is safe. You know the inquisition held over me. Dennis Clary and my d—d wife murdered me. I was driven to this by the witchcraft of my d—d family.—The first assault I made, they shut me up in a mad-house for life, and thought they had smashed my figure-head. I got out from that slaughter-house, and then Clary and my wife still pursued me. I prefer death to being locked up in yon cell. Life and liberty are no use to me. I was hunted like a wild beast in Bethlehem. I was thirteen years in England, and as many more in America, and not a man could say I injured him.— This is not an execution, but a crucifixion. When Charley Gifford (his counsel) said 'God protect you' to me, he perjured himself. Courtlandt Parker (the prosecuting attorney) sent Andy McConnell to take my life. I have faith in Almighty God for forgiveness. I have injured no one, but am chased by witchcraft.

"Almighty God! look down upon me, and see what I suffer, and forgive me. But, O God of justice, take vengeance on my d—d family, who have bewitched me! Jesus Christ, look down from heaven, and see how they murder people in asylums, and witness the abortions of the doctors! O, may the vengeance of an all-wise, all just, and powerful God fall upon them all!

"This country will be destroyed, as were Jerusalem, and Siden, and Sodom, and Gomarrah. Poole was a d—d villian to rob me of my property on Broad street. He is now in hell, for he died a raving maniac.— Turbett's wife told me she would stand by me, even if I killed her, and so she did; for I heard her voice the first thing this morning, saying: '*I have you now!*' May God have mercy on my soul!"

6. As the last link in our chain of *facts* we adduce the following "record of the post mortem appearances in the brain of Patrick Maude," the original copy of which with signatures was kindly furnished to us by Dr. Grant.

"The examination was made five hours after the execution. Present— Drs. Dougherty, Coles, Grant, Cross, Richmond, Mill.

The face was pale, not ecchymosed nor congested; the pupils, which were dilated immediately after death, had contracted in a marked degree; there was a deep discolored indentation on the right side of the neck made by the rope, the knot having rested under the ear of the opposite side.— The skull, in shape, was compressed laterally. There was no unusual amount of congestion in the vessels of the scalp. On removing the calvaria it was found thick and condensed, as in a state of eburnation. The dura-mater was adherent very generally to the arachnoid membrane, and attached to its inner surface on the right side, over the middle lobe, about an inch and one eighth from the mesial line, was a fibrocartilaginous tumor, about the size of a pea, which fitted into an indentation on the surface of the brain. There was considerable sub-arachnoid effusion and apparent thickening of the arachnoid membrane. The Pacchionian bodies, were, as observed in many cases of insanity, enlarged, and had by their pressure produced absorption of the overlaying dura mater, rendering them perceptible both to touch and sight the moment the calvaria was removed the substance of the brain was apparently in a normal state.

(Signed)

Alex. N. Dougherty,
G. Grant,
A. M. Mills,
J. A. Cross,
Jno. B. Richmond.

We have the dura mater of Maude, with the tumor attached, together with portions of the brain ourselves, and as far as this goes, can fully corroborate the above. The tumor at its base was at least one-fifth of an inch in diameter, of a conical shape, and from base to top measuring about one-fourth of an inch.—The pia mater and arachnoid in those portions of the brain which we saw, presented an opaline appearance. The post-mortem examination leaves no doubt that Maude's brain was in an abnormal condition.

The foregoing *facts* inevitably lead to the following conclusions:

1. That Maude was suffering from disease of the brain, manifesting itself in sensual and intellectual hallucinations; that he believed these hallucinations to be real, and hence was insane.

2. That his chief hallucination consisted in the firm belief of a conspiracy and an inquisition instituted against him, and the firm conviction that certain persons were practicing witchcraft upon him; that this hallucination dated back prior to his *first* trial; and that he looked upon his sister whom he murdered, as one of the leaders in that conspiracy.

3. That the crime for which he was executed was committed by him under the influence of his hallucinations. But as

4. An act commitied under the influence of sensual or intellectual hallucinations, precludes the exercise of the free will, and is never looked upon as the result of free mental agency, and hence not punishable (except in drunkenness, where the hallucinatory state has been brought on voluntarily)—hence

5. The execution of Maude—*the insane*—must be looked upon—harsh as the expression may sound—as a judical murder.

These conclusions necessarily lead to the question, why was the plea (or we should say the *fact*) of insanity unavailing in the case of Maude?

The first and foremost reason for this, is to be found in the application of the legal dogma, that a man, knowing right from wrong, is to be held responsible for his acts, though he be insane. This legal dogma is still sustained in all our codes, though long abolished in France and Germany as incorrect, though it has been demonstrated to be false by the highest authorities and men of the largest experience, and has been disregarded in many cases by judges here as well as in England. It is shown to be false by the fact that insane men, not only exceptionally, but in the immense majority of cases, are capable of distinguishing between right and wrong, not only in the abstract, but often even in regard to the particular act of violence for which they are tried. It is not necessary to enumerate the many cases in which the most enlightened judges have set aside this test, and where persons have been acquitted on high criminal charges, as *insane*, though they "knew right from wrong." The books abound in such cases.

From the extracts of the medical testimony which we have given, the reader has seen that, while all medical witnesses testify, more or less strongly, *that Maude was really suffering under insane delusions or hallucination*, they are also unanimous in expressing their belief *that Maude knew right from wrong.* This of course, under the dispensation of the right and wrong test, was the guide-post pointing to the gallows. In vain do we look in the medical testimony for some *qualification* to the affirmative answer, when the question was asked, whether Maude knew right from wrong; some statement on the part of the medical witnesses to the effect *that Maude knew right from wrong*, "as most insane people do," a scientific *fact*, so well established, that Maude was entitled to its benefit, and the omission of which renders his trial incomplete.

It may be objected, that it is not for the medical witness to volunteer testimony; that it is not his business to state the *fact*, "that most insane people know right from wrong," but that his simple duty is to answer "*yes*" or "*no.*" We do not wish to argue this point any further than to state our conviction, that, while the physician is bound to state the whole truth, the whole truth is not conveyed to a jury, without the qualification that the knowledge of right or wrong is no test of sanity. This statement made, let judge and jury take care of it.

Another reason which contributed to the failure of the plea of insanity in Maude's case, was the introduction of that unfortunate term, "*partial insanity.*" "Partial insanity" is a convenient term to signify the limited symptoms of the disease, and with this meaning may be safely used among physicians. But on the witness stand, the physician is not asked whether *all the symptoms* were present in their most acute form, but whether there were sufficiently marked symptoms to justifiy a diagnosis.

Some time ago a case happened where a Quack was prosecuted for having inoculated small-pox in several persons, instead of vaccinating. What would we think of a physician who testified that one of the patients had only "*partial small-pox*," because in his case there were but a few pustules. *The manifestation, the symptoms* were partial, not the *disease.* A man *insane*, is *insane*, and the idea that the human *mind*, the functions of which are so much more *complex and depending upon each other*, as the brain of man is superior to the nervous system of the lower animals, can be totally deranged in *one particular*, and leave all the rest perfectly intact, is contrary to sound reasoning as well as experience.

However we do not wish to lengthen this paper by going into an argument. We have stated the *facts* in Maude's case, drawn our *conclusions*, and shown the *reasons*, why the plea did not avail. If the *facts* which we have stated can be proven to be *false*, the *deductions* therefrom *illogical*, let it be done. Until then we are more than ever willing to adopt the language of Dr. Chandler R. Gilman:

"The law must not continue this already too long catalogue of judicial murders. The law must not keep in her rusty armory a test of sanity, which every man who has any knowledge of the subject knows to be vain and futile; the law must not keep this relic of an unenlightened age by her, to be brought out, as whim, or chance, or the feeling of the hour may dictate, to slay those whom the Almighty in his mysterious, most mysterious Providence, has visited with a disease, compared to which all other and mere physical diseases are but as nothing. Such beings, instead of being dragged to the scaffold or thrust into the prison-house, should be hallowed by their great misery. The heathen worshipped the tree that had been struck by lightning; let not Christian men be found less easily moved to sympathy with human sorrows."—*Med. & Surg. Reporter.*

